# Immunisation of two rodent species with new live-attenuated mutants of *Yersinia pestis* CO92 induces protective long-term humoral- and cell-mediated immunity against pneumonic plague

**DOI:** 10.1038/npjvaccines.2016.20

**Published:** 2016-10-13

**Authors:** Bethany L Tiner, Jian Sha, Yingzi Cong, Michelle L Kirtley, Jourdan A Andersson, Ashok K Chopra

**Affiliations:** 1 Department of Microbiology and Immunology, University of Texas Medical Branch, Galveston, TX, USA; 2 Institute for Human Infections and Immunity, University of Texas Medical Branch, Galveston, TX, USA; 3 Department of Pathology, University of Texas Medical Branch, Galveston, TX, USA; 4 Sealy Center for Vaccine Development and World Health Organization Collaborating Center for Vaccine Research, University of Texas Medical Branch, Galveston, TX, USA; 5 Center for Biodefense and Emerging Infectious Diseases, University of Texas Medical Branch, Galveston, TX, USA

## Abstract

We showed recently that the live-attenuated Δ*lpp* Δ*msbB* Δ*ail* and Δ*lpp* Δ*msbB::ailL2* mutants of *Yersinia pestis* CO92 provided short-term protection to mice against developing subsequent lethal pneumonic plague. These mutants were either deleted for genes encoding Braun lipoprotein (Lpp), an acetyltransferase (MsbB) and the attachment invasion locus (Ail) (Δ*lpp* Δ*msbB* Δ*ail*) or contained a modified version of the *ail* gene with diminished virulence (Δ*lpp* Δ*msbB::ailL2*). Here, long-term immune responses were first examined after intramuscular immunisation of mice with the above-mentioned mutants, as well as the newly constructed Δ*lpp* Δ*msbB* Δ*pla* mutant, deleted for the plasminogen-activator protease (*pla*) gene instead of *ail*. *Y. pestis-*specific IgG levels peaked between day 35 and 56 in the mutant*-*immunised mice and were sustained until the last tested day 112. Splenic memory B cells peaked earlier (day 42) before declining in the Δ*lpp* Δ*msbB::ailL2* mutant-immunised mice while being sustained for 63 days in the Δ*lpp* Δ*msbB* Δ*ail* and Δ*lpp* Δ*msbB* Δ*pla* mutant-immunised mice. Splenic CD4^+^ T cells increased in all immunised mice by day 42 with differential cytokine production among the immunised groups. On day 120, immunised mice were exposed intranasally to wild-type (WT) CO92, and 80–100% survived pneumonic challenge. Mice immunised with the above-mentioned three mutants had increased innate as well as CD4^+^ responses immediately after WT CO92 exposure, and coupled with sustained antibody production, indicated the role of both arms of the immune response in protection. Likewise, rats vaccinated with either Δ*lpp* Δ*msbB* Δ*ail* or the Δ*lpp* Δ*msbB* Δ*pla* mutant also developed long-term humoral and cell-mediated immune responses to provide 100% protection against developing pneumonic plague. On the basis of the attenuated phenotype, the Δ*lpp* Δ*msbB* Δ*ail* mutant was recently excluded from the Centers for Disease Control and Prevention select agent list.

## Introduction

There has been a rise in the number of human plague cases globally resulting in the categorisation of *Yersinia pestis*, the aetiological agent of the highly fatal pneumonic plague, as a re-emerging pathogen by the World Health Organization.^1^ The progression of pneumonic plague is very rapid after first appearance of the symptoms in humans, and the case fatality rate approaches 100%, if the antimicrobial treatment is delayed.^2–4^ Unfortunately, antibiotic-resistant *Y. pestis* strains have been isolated from plague patients and/or engineered for bioweaponization,^4^ which is concerning as *Y. pestis* is classified by the Centers for Disease Control and Prevention (CDC) as a Tier-1 select agent.^4^ The optimal strategy for protection against this deadly disease would be through vaccination; however, there are currently no Food and Drug Administration (FDA)-licensed plague vaccines available in the United States.^5–7^

Live-attenuated vaccines promote both humoral- and cell- mediated immune responses making them the optimal option to protect humans against pneumonic plague.^5,8^ The various live-attenuated *Y. pestis* EV76 vaccine strains, which lack the pigmentation locus (*pgm*) required for iron acquisition, have been used in plague endemic parts of the world due to vaccine strains’ ability to provide protection against both bubonic and pneumonic plague.^5^ Unfortunately, these EV76-based vaccines are not FDA approved because of high reactogenicity and these strains are not genetically uniform.^9^ In addition, Δ*pgm* mutants of *Y. pestis* cause fatal infection in individuals with diseases such as hemochromatosis.^10,11^

Subunit plague vaccines, mainly composed of two immunogens, namely F1 capsular antigen and a type III secretion system component and effector low calcium response V antigen (LcrV), are generally protective across various animal species^5,8,12–18^ but such vaccines largely generate a humoral immune response. Furthermore, F1-LcrV-based vaccines would not be ideal against infection with *Y. pestis* strains devoid of capsule or those harbouring variants of LcrV with diverged amino acid sequences.^19–22^

Therefore, our recent efforts to develop novel live-attenuated vaccines led to the deletion and/or modification of the genes encoding Braun lipoprotein (Lpp), an acetyltransferase (MsbB), the attachment invasion locus (Ail) and the plasminogen-activator protease (Pla).^23–26^ Lpp activates toll-like receptor (TLR)-2 leading to pro-inflammatory cytokine production and septic shock.^27–30^ MsbB modifies lipopolysaccharide (LPS) resulting in its increased biological potency.^26,31–35^ Ail is an outer membrane protein with extracellular loop 2 (L2) reported to be responsible for Ail-mediated bacterial serum resistance and adherence/invasion to the host cells.^25,36–43^ Pla facilitates bacterial dissemination during bubonic and pneumonic plague as well as contributes to intracellular survival of *Y. pestis* in macrophages.^24,44^

Recently, our laboratory generated three live-attenuated mutant strains of *Y. pestis* CO92. The Δ*lpp* Δ*msbB* Δ*ail* triple mutant was shown to be safe and highly immunogenic.^23,25^ However, as Ail also has immunogenic potential,^45^ the corresponding virulence-associated amino acid residues in L2 of the *ail* gene were mutated generating the Δ*lpp* Δ*msbB::ailL2* mutant of CO92.^25^ Immunisation of mice with two doses of either Δ*lpp* Δ*msbB* Δ*ail* or the Δ*lpp* Δ*msbB::ailL2* mutant *via* the intramuscular (i.m.) route triggered robust humoral and cellular immune responses. Such vaccinated mice were 100% protected when challenged 21 days after the second immunisation with high pneumonic challenge doses (70–92 LD_50_) of wild-type (WT) CO92, indicating these vaccines were capable of providing short-term protection.^25^ We also developed a Δ*lpp* Δ*pla* double mutant of CO92, and mice immunised with this double mutant developed protective immunity against subsequent pneumonic challenge.^24^ Studies have shown that deletion of the *msbB* gene from *Y. pestis* EV76 strain modulated major immunoreactive antigens,^46^ and that the Δ*lpp* Δ*msbB* double mutant was significantly more attenuated compared with the single mutants.^26^ Therefore, we deleted *msbB* gene from the Δ*lpp* Δ*pla* double mutant to improve immunogenicity and safety of the Δ*lpp* Δ*msbB* Δ*pla* triple mutant.

It is imperative that a successful plague vaccine should generate long-term immunity in immunised animals. Thus, it is essential to examine if the newly created Δ*lpp* Δ*msbB* Δ*pla* mutant as well as the Δ*lpp* Δ*msbB* Δ*ail* and Δ*lpp* Δ*msbB::ailL2* mutants have the ability to elicit protective long-term humoral- and cell-mediated immune responses, which formed the basis of this study. To authenticate our data, we used both mouse and rat models of pneumonic plague.

## Results

### Attenuation in virulence of the newly generated Δ*lpp* Δ*msbB* Δ*pla* mutant of *Y. pestis* CO92

**Figure 1:**
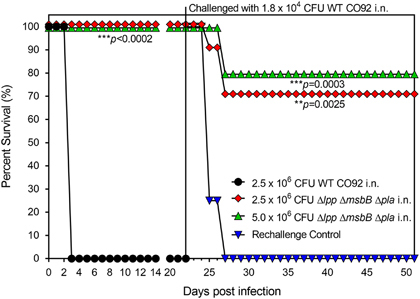
Survival analysis and protection conferred by vaccination of mice with high doses of the Δ*lpp* Δ*msbB* Δ*pla* mutant of *Y. pestis* CO92 in a pneumonic plague mouse model. Female Swiss-Webster mice (*n*=5 per group) were exposed by the i.n. route with one dose of 2.5×10^6^ CFU or 5×10^6^ CFU of the Δ*lpp* Δ*msbB* Δ*pla* mutant on day 0. Mice infected with the 2.5×10^6^ CFU of WT CO92 *luc2* served as a control. Surviving mice were then rechallenged i.n. on day 22 with 1.8×10^4^ CFU (36 LD_50;_ 1 LD_50_≅500 CFU) of WT CO92 *luc2* strain.^24^ Age-matched naive mice were used as a rechallenge control. The *P* values were calculated using Kaplan–Meier analysis with log-rank (Mantel–Cox) test and were in comparison to naive controls.

To gauge the extent of attenuation, mice (*n*=5 per group) were infected by the intranasal (i.n.) route with 2.5×10^6^ colony forming units (CFU) or 5×10^6^ CFU doses of the WT CO92 or the Δ*lpp* Δ*msbB* Δ*pla* mutant (representing 5,000 and 10,000 LD_50_ of the WT bacterium).^24^ Although mice inoculated with the WT CO92 died by day 3 post infection (p.i.), all mice infected with the Δ*lpp* Δ*msbB* Δ*pla* mutant survived with no clinical signs of the disease such as ruffled fur, hunch back and lethargy (Figure 1). On day 22, the surviving mice as well as the age-matched naive controls were exposed i.n. to 1.8×10^4^ CFU dose of WT CO92 (36 LD_50_). All of the naive mice succumbed to infection by day 27 (5 days p.i.). Animals receiving the higher immunisation dose of the Δ*lpp* Δ*msbB* Δ*pla* mutant had 80% survival after WT CO92 infection; although dropping to 70% at the lower vaccination dose (Figure 1).

### Evaluation of long-term humoral immunity in mice after immunisation with live-attenuated mutants of *Y. pestis* CO92

To further gauge vaccine potential of these three mutants (i.e. Δ*lpp* Δ*msbB* Δ*ail*, Δ*lpp* Δ*msbB::ailL2* and Δ*lpp* Δ*msbB* Δ*pla*), we used the optimal vaccination regimen,^25^ which utilised two i.m. doses (2.5×10^6^ CFU/dose, 21 days apart). A recent study also indicated that parental immunisation can lead to protective mucosal immunity, by yet unidentified mechanism(s).^47^ Using the above-mentioned vaccination protocol, 100% survivability was noted with no clinical signs of disease in immunised mice, irrespective of the mutant used, up to 120 days after the initial immunisation.

**Figure 2:**
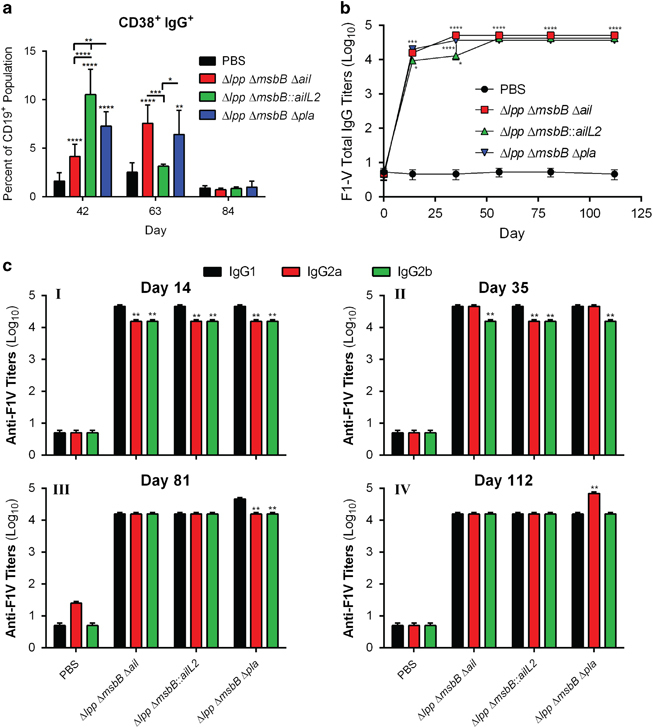
Long-term humoral immune responses in mice immunised with live-attenuated mutants of *Y. pestis* CO92. Mice (*n*=5 per group/time point) were immunised by the i.m. route with two doses (2×10^6^ CFU/dose) of Δ*lpp* Δ*msbB* Δ*ail*, Δ*lpp* Δ*msbB::ailL2* or the Δ*lpp* Δ*msbB* Δ*pla* mutants on days 0 and 21. Mice injected i.m. with PBS served as controls (*n*=3 per time point). (**a**) Spleens were harvested from mice on days 42, 63 and 84. Splenocytes were stained and analysed by Flow cytometry. Per cent CD38^+^ IgG^+^ (memory B-cell markers) expressing CD19^+^ B cells were calculated using FlowJo. Two-way ANOVA with Tukey *post hoc* was utilised for statistical analysis. (**b**) Sera were collected from mice on days 0, 14, 35, 56, 84 and 112 after first immunisation. Specific *Y. pestis* IgG was measured using ELISA against the F1-V antigen. Each time point was statistically analysed using a One-way ANOVA with Tukey *post hoc.* (**c**) Specific *Y. pestis* IgG isotypes to F1-V antigen in sera collected on day 14 (I), 35 (II), 81 (III) and 112 (IV) were measured by ELISA using isotyping specific secondary antibodies. Each time point was analysed using a two-way ANOVA with Tukey *post hoc.* *P<0.05, **P<0.01, ***P<0.001 and ****P<0.0001 as compared with PBS-injected controls. Horizontal and vertical bars represented differences between groups.

To determine long-term humoral immune response generated by the three live-attenuated vaccine strains, splenocytes were harvested from mice (*n*=3–5 per group per time point) on days 42, 63 and 84 after the first immunisation. The total CD19^+^ B-cell population was similar across all groups of mice on all days examined (~30% of the live cell population) based on flow cytometry. On day 42, mice immunised with Δ*lpp* Δ*msbB::ailL2*, Δ*lpp* Δ*msbB* Δ*pla* or the Δ*lpp* Δ*msbB* Δ*ail* mutant exhibited significantly increased CD19^+^ CD38^+^ IgG^+^ memory B-cell populations (10.5, 7.3 and 4.2%, respectively) in the spleen compared with animals injected with phosphate-buffered saline (PBS) (1.6%) (Figure 2a). By day 63, this population decreased in mice immunised with the Δ*lpp* Δ*msbB::ailL2* mutant (3.2%), which was comparable to the population observed in control (PBS) mice (Figure 2a). In comparison, mice immunised with Δ*lpp* Δ*msbB* Δ*ail* or Δ*lpp* Δ*msbB* Δ*pla* mutants continued to show increased or similar population of CD19^+^ CD38^+^ IgG^+^ memory B cells (7.6 and 6.4%, respectively) on day 63. By day 84, these populations decreased in all of the immunised groups of mice, which were similar to those in the PBS-injected mice (~0.7–1%) (Figure 2a).

Sera were collected from all mice on days 14, 35, 56, 81 and 112 after the first vaccination for measuring antigen-specific antibody responses (Figure 2b). On day 14, mice immunised with Δ*lpp* Δ*msbB* Δ*ail* or Δ*lpp* Δ*msbB* Δ*pla* mutants exhibited increased IgG antibody titres (Geometric Mean IgG titres of 15,625) against *Y. pestis* F1-V fusion antigen, representing capsular (F1) and low calcium response V (LcrV) antigens, compared with mice vaccinated with the Δ*lpp* Δ*msbB::ailL2* mutant (titre of 9,365). By day 35, a boost in the IgG antibody titres (46,875) occurred after the second immunisation (given on day 21) when mice were immunised with Δ*lpp* Δ*msbB* Δ*ail* or Δ*lpp* Δ*msbB* Δ*pla* mutants. On the contrary, a boost to the peak IgG antibody titres (46,875) in the Δ*lpp* Δ*msbB::ailL2* mutant-vaccinated mice was attained by day 56. These antibody titres remained at similar high levels until day 112 in all of the immunised groups of mice.

A significantly higher IgG1 over IgG2a/b antibody titres were noted in all of the immunised mice on day 14 (Figure 2c-I). After the second vaccine dose, and on days 35 and 56, mice immunised with the Δ*lpp* Δ*msbB* Δ*ail* and Δ*lpp* Δ*msbB* Δ*pla* mutants had balanced Th1-based IgG2a and Th2-based IgG1 antibody responses, whereas mice vaccinated with the Δ*lpp* Δ*msbB::ailL2* mutant continued to have higher IgG1 over IgG2a titres (Figure 2c-II; data not shown for day 56). By day 81, balanced Th1-based IgG2a and Th2-based IgG1 antibody titres were observed in mice immunised with the Δ*lpp* Δ*msbB* Δ*ail* or the Δ*lpp* Δ*msbB::ailL2* mutant, whereas the Δ*lpp* Δ*msbB* Δ*pla* mutant-vaccinated mice exhibited significantly higher IgG1 titres compared with IgG2a/b titres (Figure 2c-III). By day 112, mice immunised with the Δ*lpp* Δ*msbB* Δ*ail* or the Δ*lpp* Δ*msbB::ailL2* mutant maintained balanced Th1-based IgG2a and Th2-based IgG1 antibody titres. However, Δ*lpp* Δ*msbB* Δ*pla* mutant-vaccinated mice possessed significantly higher IgG2a titres compared with IgG1 antibody titres (Figure 2c-IV).

**Figure 3:**
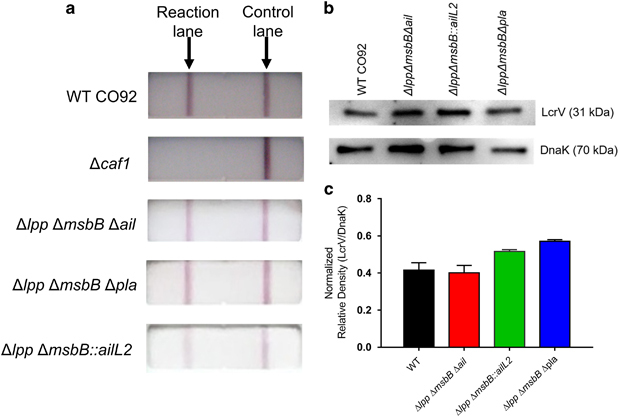
Production of LcrV and F1 by the live-attenuated mutants of *Y. pestis* CO92. To detect F1 production, 100 μl of each 37 °C grown bacterial cultures (1×10^7^ CFU) was analysed with the F1 antigen capture-based dipstick, and WT CO92 as well as its F1 minus mutant (Δ*caf1*) were used as controls (**a**). To detect LcrV production, bacterial cultures were first grown at 37 °C in the presence of 5 mmol/l EGTA to activate T3SS. Cell pellets were then subjected to western blot analysis with specific anti-LcrV antibodies, and the antibody to DnaK was employed as a control for the sample loading (**b**). The density of LcrV on the immunoblot for each lane was normalised to that of its corresponding DnaK immunoblot and presented as a bar graph (**c**).

Overall, all immunised mice maintained high levels of IgG1, IgG2a and IgG2b antibody titres over the duration of the experiment, albeit some skewing of Th1- and Th2-based immune responses occurred. Importantly, all three aforementioned mutants were metabolically active as no apparent alterations were observed in the production of both F1 and LcrV compared with the WT CO92 based on the F1 antigen capture-based dipstick and Western blot analyses (Figure 3).^23,25^

### Long-term cell-mediated immunity after immunisation of mice with live-attenuated mutants of *Y. pestis* CO92

**Figure 4:**
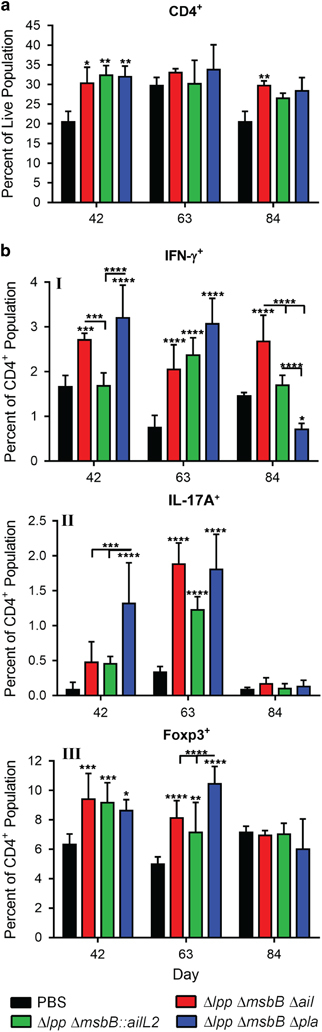
Long-term cell-mediated immune responses in mice immunised with live-attenuated mutants of *Y. pestis* CO92. Mice (*n*=5 per group/time point) were immunised by the i.m. route with two doses (2×10^6^ CFU/dose) of Δ*lpp* Δ*msbB* Δ*ail*, Δ*lpp* Δ*msbB::ailL2* or the Δ*lpp* Δ*msbB* Δ*pla* mutants on days 0 and 21. Mice were injected i.m. with PBS to serve as controls (*n*=3 per time point). Spleens were harvested from mice on days 42, 63 and 84. Splenocytes were stained and analysed by Flow cytometry. (**a**) Total per cent of CD4^+^ (T-helper cell marker) expressing cells was calculated using FlowJo. (**b**) Per cent of IFN-γ (I; Th1 marker), IL-17A (II; Th17 marker) and Foxp3 (III; T_reg_ marker) expressing CD4^+^ cells was calculated. Two-way ANOVA with Tukey *post hoc* was utilised for determining statistical significance. *P<0.05, **P<0.01, ***P<0.001 and ****P<0.0001 as compared with PBS-injected controls. Horizontal bars represent differences between the indicated groups.

To examine T cell-mediated immune responses after immunisation, splenocytes were isolated from mice and stained for T cell-specific markers on days 42, 63 and 84. By day 42, all immunised mice had statistically significant increased population of CD4^+^ cells in the spleen (Figure 4a). On day 84, although the increasing pattern still continued in all three mutant-immunised mice as compared with mice injected with PBS (20.5%), only mice vaccinated with the Δ*lpp* Δ*msbB* Δ*ail* mutant had statistically significant higher CD4^+^ population (29.7%). The CD4^+^ population was 26.5 and 28.4% in mice immunised with Δ*lpp* Δ*msbB::ailL2* and Δ*lpp* Δ*msbB* Δ*pla* mutants, respectively (Figure 4a).

To further evaluate cell-mediated immune responses in vaccinated mice, T cells were stained for selected cytokines or transcription factors. On days 42 and 63, interferon (IFN)-γ^+^ CD4^+^ cell population was significantly increased in mice immunised with the Δ*lpp* Δ*msbB* Δ*ail* mutant (~2–2.7%) or the Δ*lpp* Δ*msbB* Δ*pla* mutant (~3.1–3.2%) compared with PBS-injected mice (~0.7–1.7%) (Figure 4b-I). On day 84, although the IFN-γ^+^ CD4^+^ cell population remained significantly high in mice immunised with the Δ*lpp* Δ*msbB* Δ*ail* mutant (2.7%), this cell population decreased in Δ*lpp* Δ*msbB* Δ*pla* mutant-immunised mice (0.7%) (Figure 4b-I). The IFN-γ^+^ CD4^+^ cell population in mice vaccinated with the Δ*lpp* Δ*msbB::ailL2* mutant (~1.8%) was comparable to that of PBS-injected mice (~1.7%) on day 42, increasing significantly on day 63 (~2.4%). However, it dropped to a level similar to that in the PBS-injected mice on day 84.

IL-17A^+^ CD4^+^ cell population increased in all immunised mice on day 42 (~0.5–1.3%) compared with PBS-injected mice (0.003%) (Figure 4b-II); however, only mice immunised with the Δ*lpp* Δ*msbB* Δ*pla* mutant reached statistical significance and had the highest IL-17A^+^ CD4^+^ population compared with all other groups. Importantly, the level of IL-17A^+^ CD4^+^ cells were further elevated on day 63 in all of the immunised mice (~1.2–1.9%) compared with that on day 42, and was significant higher than that of the PBS-injected mice (~0.3%). By day 84, this subset of population in the immunised mice returned to that of the PBS-injected mice. Interestingly, Foxp3^+^ CD4^+^ cell population increased significantly in all immunised mice on day 42 (~8.6–9.4%) compared with PBS-injected mice (6.3%) (Figure 4b-III). On day 63, these levels remained elevated (~7.2–10.5%) compared with those in naive mice (~5%). By day 84, this subset of cell population in vaccinated mice returned to that of PBS-injected mice.

### Evaluation of long-term protection against pneumonic plague provided by immunisation of mice with live-attenuated mutants of *Y. pestis* CO92

**Figure 5:**
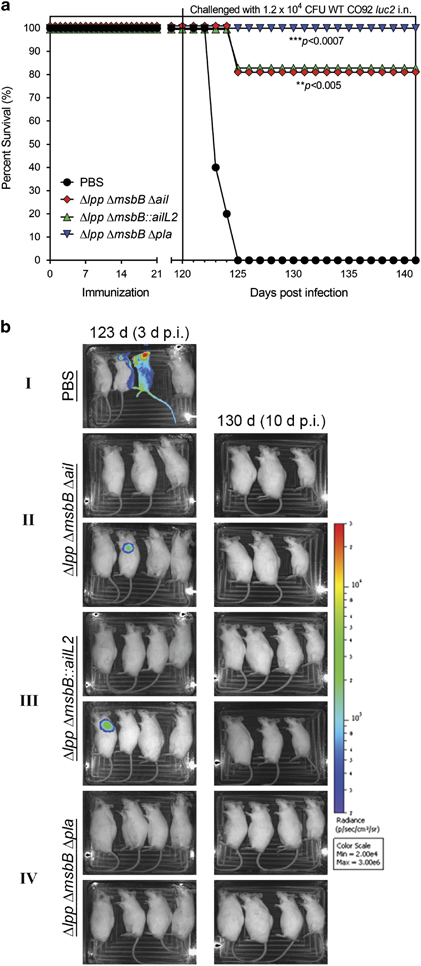
Survival analysis of immunised mice after exposure to WT *Y. pestis* CO92 in a pneumonic plague model. Mice (*n*=5–6 per group) were immunised by the i.m. route with two doses (2×10^6^ CFU/dose) of Δ*lpp* Δ*msbB* Δ*ail*, Δ*lpp* Δ*msbB::ailL2* or the Δ*lpp* Δ*msbB* Δ*pla* mutants on days 0 and 21. Mice were injected i.m. with PBS to serve as controls (*n*=5 per group). (**a**) Mice were exposed i.n. on day 120 with 1.2×10^4^ CFU (24 LD_50;_ 1 LD_50_≅500 CFU)^24^ of the WT CO92 *luc2* strain (with luciferase gene). The *P* values were calculated using the Kaplan–Meier analysis with log-rank (Mantel–Cox) test and were in comparison to the naive control. (**b**) Bioluminescence imaging of mice. Surviving mice after i.n. exposure with 1.2×10^4^ CFU of WT CO92 *luc2* strain were imaged on days 123 and 130 (days 3 and 10 p.i.). The mouse at the extreme right side in each panel was uninfected naive animal to serve as an imaging control. The bioluminescent scale is within the figures and ranges from most intense (red) to least intense (violet).

On day 120, mice were challenged *via* the i.n. route with 1.2×10^4^ CFU dose (24 LD_50_) of the WT CO92 *luc2* strain (with the luciferase gene) to mimic the pneumonic plague.^23–26,48^ The PBS-injected mice succumbed to infection by day 125 (5 days p.i.) (Figure 5a). Two of these five mice died prior to imaging for bioluminescence. The image in Figure 5b-I (PBS) showed that two of the three remaining control mice were positive for bioluminescence on day 123 (3 days p.i.). One animal with the strongest bioluminescence died immediately after the imaging, whereas the other two with somewhat weaker or negative bioluminescence died on day 4 and 5 p.i., respectively. The mouse on the extreme right side in the panel PBS represented an uninfected control for imaging. One animal from each of the Δ*lpp* Δ*msbB* Δ*ail* and Δ*lpp* Δ*msbB::ailL2* mutant-immunised groups were positive for bioluminescence, and the infection was confined to the lungs (Figure 5b-II and III). These two mice succumbed to infection on day 125 (5 days p.i.), resulting in 80% animal survival (Figure 5a). The remaining mice in these groups survived and were negative for bioluminescence on day 10 after challenge, thus suggesting clearance of WT CO92 before day 10. All of the mice immunised with the Δ*lpp* Δ*msbB* Δ*pla* mutant survived exposure to WT CO92, and they were negative for bioluminescence on both days 3 and 10 p.i. (Figures 5a, b-IV).

### Evaluation of the immediate innate immune response of vaccinated mice after exposure to WT *Y. pestis* CO92 in a pneumonic plague model

**Figure 6:**
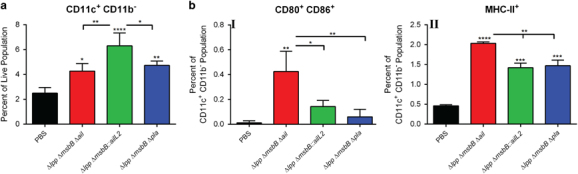
Innate immune responses in mutant-immunised mice after exposure to WT *Y. pestis* CO92 in a pneumonic plague model. Mice were immunised by the i.m. route with two doses (2×10^6^ CFU/dose) of Δ*lpp* Δ*msbB* Δ*ail*, Δ*lpp* Δ*msbB::ailL2* or the Δ*lpp* Δ*msbB* Δ*pla* mutants on days 0 and 21. Mice i.m. injected with PBS served as controls. On day 120, all mice were exposed i.n. to 1.2×10^4^ CFU (24 LD_50;_ 1 LD_50_≅500 CFU)^24^ of the WT CO92 *luc2* strain. Spleens were harvested from mice (*n*=3–4 per group) on day 124 (days 4 p.i.). Splenocytes were stained and analysed by Flow cytometry. (**a**) Total per cent of CD11c^+^ CD11b^−^ (resident DC markers) expressing cells was calculated using FlowJo. (**b**) Per cent of CD80^+^ CD86^+^ (I; activation markers) and MHC-II^+^ (II) expressing DCs were also calculated. Two-way ANOVA analysis with Tukey *post hoc* was utilised for determining statistical significance. *P<0.05, **P<0.01, ***P<0.001 and ****P<0.0001 as compared with PBS-injected controls. Horizontal bars represent differences between the indicated groups.

On day 120, both mutant-immunised- and PBS-injected control mice were exposed to 24 LD_50_ of WT CO92 *luc2* strain. On day 124 (4 days p.i.), spleens were harvested from a subset of the challenged mice (*n*=3–4 per group) to determine innate immune cell response subsequent to the challenge. The total number of CD11c^+^ CD11b^−^ resident dendritic cells (DCs) was maximally increased in mice immunised with the Δ*lpp* Δ*msbB::ailL2* mutant (6.3%) compared with the other two groups of vaccinated (with Δ*lpp* Δ*msbB* Δ*ail* (4.3%) or Δ*lpp* Δ*msbB* Δ*pla* (4.7%) mutant) mice (Figure 6a). Importantly, the DC numbers significantly increased in all immunised mice compared with mice that were injected with PBS and exposed to the WT CO92 *luc2* strain (2.5%) (Figure 6a).

The total number of CD80^+^ CD86^+^ cells, which represented activated CD11c^+^ CD11b^−^ DC populations, was maximally increased in mice immunised with the Δ*lpp* Δ*msbB* Δ*ail* mutant followed by the Δ*lpp* Δ*msbB::ailL2* and Δ*lpp* Δ*msbB* Δ*pla* mutants subsequent to the WT CO92 exposure (Figure 6b-I). These activated DC cell numbers were in comparison to mice injected with PBS and exposed to WT CO92. Likewise, MHC-II expression was highest in the CD11c^+^ CD11b^−^ cell population isolated from mice vaccinated with the Δ*lpp* Δ*msbB* Δ*ail* mutant followed by that of Δ*lpp* Δ*msbB::ailL2* or Δ*lpp* Δ*msbB* Δ*pla* mutants when compared with mice injected with PBS and exposed to WT CO92 (Figure 6b-II).

### Evaluation of cytokine producing CD4^+^ T cells in immunised mice after exposure to WT *Y. pestis* CO92 in a pneumonic plague model

Similar to the DC staining above, the isolated splenocytes were stained for T cell markers to determine immune recall response after exposure to WT CO92, and an additional time point on day 141 (21 days p.i.) was also included. For the 124-day time point, spleens from PBS-injected mice with and without exposure to WT CO92 were used as controls. As all PBS-injected mice, exposed to WT CO92 succumbed to infection by day 141 (21 days p.i.), we only used PBS-injected mice not exposed to WT CO92 as controls for this time point.

**Figure 7:**
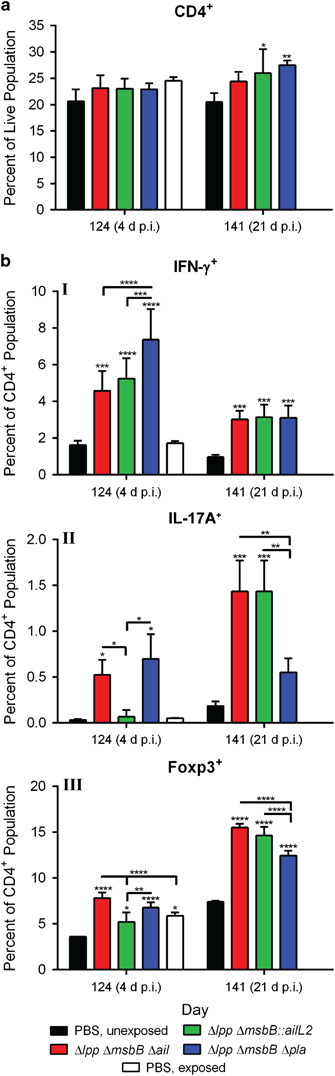
Cell-mediated immune responses in mutant-immunised mice after exposure to WT *Y. pestis* CO92 in a pneumonic plague model. Mice were immunised by the i.m. route with two doses (2×10^6^ CFU/dose) of Δ*lpp* Δ*msbB* Δ*ail*, Δ*lpp* Δ*msbB::ailL2* or the Δ*lpp* Δ*msbB* Δ*pla* mutants on days 0 and 21. Mice were exposed i.n. on day 120 with 1.2×10^4^ CFU (24 LD_50;_ 1 LD_50_≅500 CFU)^24^ of the WT CO92 *luc2* strain. Mice i.m. injected with PBS and then either exposed or not exposed to WT CO92 *luc2* served as controls. Spleens were harvested from surviving mice (3–5 per group) on days 124 (4 days p.i.) and 141 (21 days p.i.). Splenocytes were stained and analysed by Flow cytometry. (**a**) Total per cent of CD4^+^ (T-helper cell marker) expressing cells was calculated using FlowJo. (**b**) Per cent of IFN-γ (I; Th1 marker), IL-17A (II; Th17 marker) and Foxp3 (III; T_reg_ marker) expressing CD4^+^ cells was calculated. Two-way ANOVA analysis with Tukey *post hoc* was utilised for determining statistical significance. *P<0.05, **P<0.01, ***P<0.001 and ****P<0.0001 as compared with PBS-injected controls. Horizontal bars represent differences between the indicated groups.

On day 124 (4 days p.i.), there were no differences in the total number of CD4^+^ cell population in any groups of the immunised and control mice after challenge with WT CO92 (Figure 7a). By day 141 (21 days p.i.), mice immunised with Δ*lpp* Δ*msbB::ailL2* or Δ*lpp* Δ*msbB* Δ*pla* mutants had increased number of total CD4^+^ population (~260–27.5%) compared with Δ*lpp* Δ*msbB* Δ*ail* mutant-immunised mice, or those which served as controls (Figure 7a).

Early after exposure to WT CO92 (day 4 p.i.), the spleens of all immunised mice, for example, with Δ*lpp* Δ*msbB* Δ*ail*, Δ*lpp* Δ*msbB::ailL2* or the Δ*lpp* Δ*msbB* Δ*pla* mutants possessed increased percentage of IFN-γ^+^ CD4^+^ cells (4.6, 5.2 and 7.4%), respectively. This was in comparison to mice injected with PBS but not exposed to WT CO92 (1.6%) as well as to those mice that were injected with PBS and exposed to WT CO92 (1.7%) (Figure 7b-I). By day 21 p.i., the percentage of IFN-γ^+^ CD4^+^ cells decreased in all surviving mice immunised with Δ*lpp* Δ*msbB* Δ*ail*, Δ*lpp* Δ*msbB::ailL2* or Δ*lpp* Δ*msbB* Δ*pla* mutants (3, 3.2 and 3.4%, respectively) (Figure 7b-I). However, this cell population was still significantly higher when compared with the control naive mice without exposure to WT CO92 (1%).

IL-17A^+^ CD4^+^ cells were increased in spleens of mice immunised with the Δ*lpp* Δ*msbB* Δ*ail* and Δ*lpp* Δ*msbB* Δ*pla* mutants compared with mice vaccinated with the Δ*lpp* Δ*msbB::ailL2* mutant or injected with PBS after exposure to WT CO92 on day 4 p.i.(Figure 7b-II). On day 141 (21 days p.i.), the percentage of IL-17A^+^ CD4^+^ cells was further increased in spleens of mice immunised with the Δ*lpp* Δ*msbB* Δ*ail* mutant, whereas maintained at a similar level in mice vaccinated with the Δ*lpp* Δ*msbB* Δ*pla* mutant compared with day 4 p.i. On the contrary, a robust increase in the population of IL-17A^+^ CD4^+^ cells was noted in mice vaccinated with the Δ*lpp* Δ*msbB::ailL2* mutant followed by exposure to WT CO92 on day 141 (21 days p.i.) (Figure 7b-II).

On day 124 (4 days p.i.), the Foxp3^+^ CD4^+^ cell population was increased in mice vaccinated with Δ*lpp* Δ*msbB* Δ*ail*, Δ*lpp* Δ*msbB::ailL2* or the Δ*lpp* Δ*msbB* Δ*pla* mutants (7.8, 5.2 and 6.8%, respectively) when compared with PBS-injected and unexposed naive control (3.6%) (Figure 7b-III). However, this difference was only significant for the Δ*lpp* Δ*msbB* Δ*ail* mutant when compared with mice that were injected with PBS and exposed to WT CO92. By day 141 (21 days p.i.), all surviving mice immunised with Δ*lpp* Δ*msbB* Δ*ail*, Δ*lpp* Δ*msbB::ailL2* or Δ*lpp* Δ*msbB* Δ*pla* mutants had increased Foxp3^+^ CD4^+^ cells (15.5, 14.7 and 12.5%, respectively), compared with control mice (7.4%) (Figure 7b-III).

### Evaluation of humoral and cell-mediated immunity in the mutant-immunised rats necessary for protection against WT *Y. pestis* CO92 challenge in a pneumonic plague model

On the basis of the mouse data presented above, both Δ*lpp* Δ*msbB* Δ*ail* and Δ*lpp* Δ*msbB* Δ*pla* mutants elicited slightly better humoral and cell-mediated immune responses than that of the Δ*lpp* Δ*msbB::ailL2* mutant. Therefore, these two mutants were further evaluated in a rat model of pneumonic plague to authenticate mouse data.

**Figure 8:**
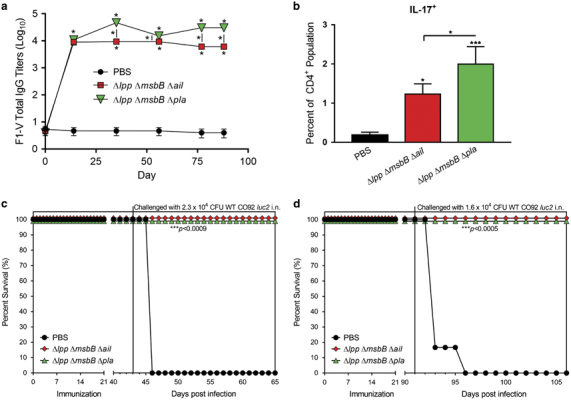
Immune response and protection in rats conferred by immunisation with mutants of *Y. pestis* CO92 against pneumonic plague. Brown Norway rats were immunized by the i.m. route with two doses (2×10^6^ CFU/dose) of Δ*lpp* Δ*msbB* Δ*ail* or the Δ*lpp* Δ*msbB* Δ*pla* mutant on days 0 and 21. Rats were injected i.m. with PBS to serve as controls. (**a**) Sera were collected from rats on days 0, 14, 35, 56, 77 and 88 after first immunization. Specific *Y. pestis* IgG was measured using ELISA against the F1-V antigen. Each time point was statistically analysed using a one-way ANOVA with Tukey *post hoc.* (**b**) Spleens were harvested from rats on days 42. Splenocytes were stained and analysed by Flow cytometry. IL-17A^+^ (Th17 marker) expressing CD4^+^ cells were calculated. Two-way ANOVA with Tukey *post hoc* was utilised for determining statistical significance. *P<0.05 and ***P<0.001 as compared with PBS-injected controls. Horizontal and vertical bars represented differences between groups. Rats were challenged via the i.n. route with WT CO92 *luc2* strain at the dose of either 2.3×10^4^ CFU (46 LD_50_) on day 43 (**c**) or 1.6×10^4^ CFU (31 LD_50_) on day 91 (**d**). The survival of rats was recorded and plotted. The *P* values were calculated using the Kaplan–Meier analysis with log-rank (Mantel–Cox) test and were in comparison to the naive control.

Similar to the mouse model, inbred Brown Norway rats were immunised with two i.m. doses (2.5×10^6^ CFU/dose, 21 days apart) of the selected mutants, and rats receiving PBS injection served as a control. Sera were collected from all rats on days 0, 14, 35, 56, 77 and 88 after the first vaccination for measuring antigen-specific antibody responses. As shown in Figure 8a, the immunised rats in both immunisation groups exhibited high IgG antibody titres (Geometric Mean IgG titres of 9,325) against *Y. pestis* F1-V fusion antigen on day 14. More specifically, in rats immunised with the Δ*lpp* Δ*msbB* Δ*ail* mutant, the IgG antibody titres remained plateaued until day 56 with a slight decrease on day 77 and then maintained at the same level to the last examining day (day 88). On the other hand, by day 35, a boost in the IgG antibody titres (46,875) occurred in rats immunised with the Δ*lpp* Δ*msbB* Δ*pla* mutant and remained high until day 88.

To evaluate CD4^+^ T cell-mediated immune responses in vaccinated rats, splenocytes (*n*=3 per group) were isolated on day 42. Although all groups of rat (both immunised and PBS-injected control) had similar number of total CD4^+^ cell populations (~31.4–33.9%), significantly increased IL-17A^+^ CD4^+^ cell population was observed in both groups of immunised rats (~1.2–2%) compared with the PBS-injected control rats (0.2%) (Figure 8b). Furthermore, rats immunised with the Δ*lpp* Δ*msbB* Δ*pla* mutant had higher IL-17A^+^CD4^+^ population than that of animals vaccinated with the Δ*lpp* Δ*msbB* Δ*ail* mutant (Figure 8b). The IFN-γ^+^ CD4^+^ cell population also increased in both mutant-immunised rats (~15.5–19.9%) as compared with the PBS-injected control rats (~8.5%); however, a statistical significance was not achieved (data not shown).

To further evaluate the vaccine efficacy of the two mutants, the immunised rats (*n*=6 per group) were challenged via the i.n. route with WT CO92 *luc2* strain at the dose of either 2.3×10^4^ CFU (46 LD_50_) on day 43 to evaluate short-term protection or 1.6×10^4^ CFU (31 LD_50_) on day 91 to evaluate long-term protection. As shown in Figure 8c,d, all the PBS-injected control rats succumbed to infection. On the contrary, 100% survival was noted in rats immunised with either the Δ*lpp* Δ*msbB* Δ*ail* or the Δ*lpp* Δ*msbB* Δ*pla* mutant. Most importantly, no WT CO92 *luc2* strain were detected in the blood and organs (lungs, liver and spleen) from both groups of immunised rats 14 days after challenge for the long-term protection assessment, indicating that the challenged bacterium was cleared from the immunised rats.

## Discussion

It is crucial that a potential vaccine candidate(s) demonstrates long-term immune responses and protection. This is the first study to examine several components of the immune response generated after immunisation of mice and rats with the above three live-attenuated mutants over a period of 3–4 months.

The kinetics of memory B-cell production in *Y. pestis* EV76 vaccine strain-immunised mice has been reported.^49^ Similar to our protocol, mice were immunised i.m. with two doses (8×10^7^ CFU/dose) of EV76 (21 days apart) and splenocytes harvested periodically after immunisation.^49^ However, CD38 and IgD expressing CD19^+^ CD27^+^ population was evaluated in that study. CD27 is not considered as an appropriate marker for memory B cells in mice,^50,51^ and the lack of CD27 expression on memory B cells has been previously reported.^52^ CD38 has been widely accepted as a marker of memory B cells as antigen-specific IgG1 B cells with high CD38 expression and similar characteristics to recirculating memory B cells are apparent weeks after immunisation.^50,53^ Although IgD and IgG are produced by activated B cells, we designated memory B-cell population as CD38 and IgG producing CD19^+^ cells. By day 7, memory B-cell populations increased in EV76-immunised mice in the spleen.^49^ However, this population of cells sharply decreased by day 56.^49^ Memory B cells in the Δ*lpp* Δ*msbB::ailL2* mutant*-*immunised mice behaved similar to those in the EV76-immunised animals with increases in the CD19^+^ CD38^+^ IgG^+^ cell population on day 42 which decreased to the levels seen in naive mice by day 63 (Figure 2a). However, memory B-cell population in Δ*lpp* Δ*msbB* Δ*ail* and Δ*lpp* Δ*msbB* Δ*pla* mutant*-*immunised mice increased or remained high through day 63 and declined to levels seen in naive mice only on day 84 (Figure 2a).

In terms of antibody production, total IgG titres in the Δ*lpp* Δ*msbB::ailL2* mutant-immunised mice reached a maximal level on day 56 and then plateaued, whereas mice immunised with the other two mutants, Δ*lpp* Δ*msbB* Δ*ail* and Δ*lpp* Δ*msbB* Δ*pla*, showed maximum IgG titres by day 35 and then plateaued (Figure 2b). In contrast, the total memory B-cell population peaked at day 42 in the Δ*lpp* Δ*msbB::ailL2* mutant-immunised mice, which then decreased to levels of PBS-injected, naive mice by day 63 (Figure 2a). Interestingly, mice immunised with the other two mutants were capable of sustaining memory B-cell populations until day 63 (Figure 2a). These differences in time to peak antibody titres versus the maximum percentage of memory B-cell population in the Δ*lpp* Δ*msbB::ailL2* mutant-immunised mice (Figure 2a,b) could be attributed to low avidity of the IgG antibodies to F1-V antigens of *Y. pestis* early during vaccination before switching to high-affinity antibodies later during immunisation. Alternatively, these memory B cells from Δ*lpp* Δ*msbB::ailL2* mutant-immunised mice might be secreting less IgG per cell compared with that of the memory B cells of Δ*lpp* Δ*msbB* Δ*ail-* and Δ*lpp* Δ*msbB* Δ*pla* mutant*-*immunised mice because of a high proportion of unswitched memory B cells early during immunisation.^54^

Mice immunised i.m. with two doses (8×10^7^ CFU/dose) of EV76 strain (21 days apart) had lower total F1-specific IgG titres (10,000–15,000 on day 42).^55^ These titres decreased below 10,000 by day 70, and further declined before attaining a steady state level (1,400) by day 322.^55^ In comparison, mice vaccinated with our live-attenuated mutants at a lower dose (2×10^6^ CFU/dose) were not only able to achieve total F1-V specific IgG titres of 46,875 by day 35–56 but were able to maintain these maximum titres until day 112 (Figure 2b).

Overall, all three live-attenuated mutants stimulated long-lasting T cell-mediated immune responses capable of protecting mice from developing subsequent pneumonic plague. Our data generally indicated an increase in IFN-γ-, IL-17A- and Foxp3- expressing CD4^+^ T cells after immunisation (Figure 4). A similar trend was also noted when these mutant-immunised mice were subsequently exposed to WT CO92 in a pneumonic model (Figure 7). All immunised mice that survived WT CO92 *luc2* strain challenge rapidly cleared the bacteria (3 days p.i.) as measured by bioluminescence (Figure 5b), possibly due to increased production of IFN-γ by CD4^+^ cells (Figure 7b-I). IFN-γ promotes macrophage activation facilitating defence against bacterial pathogens,^56^ and it has been shown that IFN-γ as well as tumour necrosis factor-α are important co-determinants of antibody-mediated protection against pneumonic plague.^57^ Consequently, the increased presence of IFN-γ^+^ CD4^+^ cells in conjunction with F1-V specific neutralising antibody production could augment opsonization and clearance of *Y. pestis*.

Th17 cells are potent secretors of IL-17A, and it has recently been shown that IL-17A provides an antibody-independent heterologous protection of the host against many pathogenic bacterial infections, including *Y. pestis.*^58,59^ For example, IL-17A was induced by the intranasal immunisation of mice with the *Y. pestis* strain D27-pLpxL KIM/D27 engineered to produce *Escherichia coli* LpxL, which increases TLR-4 activation by LPS of *Y. pestis.*^60^ Thus, IL-17A contributed significantly to T cell-mediated defence against pulmonary *Y. pestis* infection.^59^ Consistent with previous reports,^58,59^ we also showed Δ*lpp* Δ*msbB* Δ*pla* mutant*-*immunised mice had the highest increase in IL-17A producing CD4^+^ cells immediately after WT CO92 exposure (Figure 7b-II) resulting in efficient clearance of the invading pathogen. Importantly, the IL-17A^+^ CD4^+^ cell population decreased in the Δ*lpp* Δ*msbB* Δ*pla* mutant*-*immunised mice 21 days post WT CO92 exposure (Figure 7b-II) signifying a possible faster resolution of inflammation, which is desirable in a potential vaccine.

In contrast, mice vaccinated with Δ*lpp* Δ*msbB* Δ*ail* or Δ*lpp* Δ*msbB::ailL2* mutants and subsequently challenged with WT CO92 had increased IL-17A producing CD4^+^ cells 21 days p.i. (Figure 7b-II). Thus, these mice would most likely had somewhat of a prolonged inflammatory response after exposure to WT CO92; however, not to an extent to cause any adverse histopathological lesions in immunised mice.^25^ Interestingly, production of IL-17A from T cells was also observed in our previous study when isolated T cells from Δ*lpp* Δ*msbB* Δ*ail* and Δ*lpp* Δ*msbB*::*ailL2* mutant-immunised mice were co-cultured with antigen-presenting cells, which had been exposed to heat-killed WT CO92.^25^

Foxp3 is a transcription factor and marker for T_reg_ cells (Foxp3^+^ CD4^+^), which are primarily responsible for dampening immune responses. Recent studies have revealed that T_reg_ cells can promote protective Th17-associated immune responses against bacterial infections.^61,62^ Interestingly, both Foxp3^+^ and IL-17A^+^ CD4^+^ cells were concurrently increased both after immunisation as well as after WT CO92 challenge (Figures 4b and 7b). However, a direct link between T_reg_ cells and Th17-based protective immune responses against *Y. pestis* still needs further investigation.

Consistent with the above-mentioned data, all mutant-immunised mice had increased DC population expressing MHC-II (Figure 6b-II), which is required for antigen presentation to CD4^+^ cells.^63^ An increasing trend in the activation of DCs was noted for all mutant-immunised mice, which attained statistical significance for the Δ*lpp* Δ*msbB* Δ*ail* mutant. Overall, our results indicated immunisation of mice with any of these mutants could successfully induce activation of an innate immune response after exposure to WT CO92. Most importantly, all three live-attenuated mutants stimulated both long-term humoral- and cell-mediated immune responses, which protected mice against exposure to highly lethal pneumonic challenge (Figure 5).

In the second animal model of pneumonic plague, both the Δ*lpp* Δ*msbB* Δ*ail* and Δ*lpp* Δ*msbB* Δ*pla* mutants also stimulated robust humoral and cell-mediated immune responses, which provided both short and long-term protection to rats (100%) against WT CO92 pneumonic challenge (Figure 8). The significantly increased IL-17A^+^ CD4^+^ population in the immunised rats further highlighted the importance of IL-17 against *Y. pestis* infection.

In addition to efficiency, safety is another important aspect for vaccine development. Recently, we have shown that both the Δ*lpp* Δ*msbB* Δ*ail* and Δ*lpp* Δ*msbB*::*ailL2* mutants are safe live-attenuated vaccine candidates due to their quick clearance from injection site by 24–48 h after vaccination, and these mice had no histopathological lesions during immunisation.^25^ Our extensive data with the Δ*lpp* Δ*msbB* Δ*ail* mutant^23,25^ led to its exclusion from the CDC select agent list. In this study, we have further demonstrated that the WT CO92 *luc2* challenge strain was efficiently cleared from the immunised mice and rats after 10–14 days post challenge as examined by using either IVIS or the plate count method. Our future goals are to obtain permission from the CDC to exclude Δ*lpp* Δ*msbB*::*ailL2* and Δ*lpp* Δ*msbB* Δ*pla* mutants from the select agent list, allowing safe production of these vaccines under biosafety level 2 precautions. We will further evaluate safety of these live-attenuated vaccine candidates in immunocompromised mice, study in more depth their immune protection mechanisms, and to finally examine efficacy of these vaccines in non-human primates.

## Materials and methods

*Y. pestis* and recombinant *E. coli* strains were grown as described by us previously.^23,24,26^ All of our studies were performed in a Tier-1 select agent facility within the Galveston National Laboratory (GNL), UTMB.

### Creation of the Δ*lpp* Δ*msbB* Δ*pla* mutant of *Y. pestis* CO92

The in-frame deletion of the *msbB* gene from the Δ*lpp* Δ*pla* double mutant of *Y. pestis* CO92 was prepared using the suicide vector pDMS197 followed by homologous recombination as described previously by our laboratory.^26^ The in-frame deletion of the *msbB* gene was confirmed by polymerase chain reaction (PCR) analysis employing specific primers^26^ as well as by DNA sequencing of the flanking regions to the *msbB* gene on the chromosome.

### Production of LcrV and F1 by the live-attenuated mutants of *Y. pestis* CO92

WT CO92 and the Δ*lpp* Δ*msbB* Δ*ail*, Δ*lpp* Δ*msbB*::*ailL2.* and Δ*lpp* Δ*msbB* Δ*pla* mutants were grown overnight in heart infusion broth (HIB) at 28 °C and then diluted 1:20 in 5 ml HIB supplemented with 5 mM EGTA. Cultures were incubated at 28 °C for 2 h and then at 37 °C (to activate the T3SS) for an additional 3 h. The cell pellets were dissolved in SDS–PAGE buffer and analysed with anti-LcrV antibodies (Santa Cruz Biotechnology, Santa Cruz, CA, USA). The anti-DnaK monoclonal antibody (Enzo Life Sciences, Boston, MA, USA) was employed for analysis and normalisation of protein concentrations in cell pellets. The density of the immunoblots was analysed by using Image Studio Lite Version 5.2 (Li-Cor, Lincoln, NE, USA). To detect F1 production, 100 μl of each 37 °C grown bacterial cultures (1×10^7^ CFU) was analysed with the F1 antigen capture-based dipstick (Plague BioThreat Alert test strips, Tetracore, Inc., Rockville, MD, USA) as we previously described.^23,25^

### Animal studies

Six-to-eight-week old, female Swiss-Webster mice were purchased from Taconic Laboratories (Germantown, NY, USA), and four-to-five-week old (50–75 g) Brown Norway female rats were purchased from Charles River (Houston, TX, USA). The animal studies were performed in the Animal Biosafety Level (ABSL)-3 facility under an approved Institutional Animal Care and Use Committee protocol.

#### Attenuation

Mice were infected by the i.n. route with one dose of 2.5×10^6^ CFU/40 μl or 5×10^6^ CFU/40 μl of the Δ*lpp* Δ*msbB* Δ*pla* mutant, and mice i.n. infected with 2.5×10^6^ CFU/40 μl of WT CO92 served as control.^23,24^ Animals were assessed for morbidity and/or mortality for twenty-two days. The surviving mice were then rechallenged by the i.n. route with 1.8×10^4^ CFU/40 μl (36 LD_50_) of the bioluminescent WT *Y. pestis* CO92 *luc2* strain.^23^ The naive mice served as rechallenge controls.

#### Immunisation

Mice were immunised by the i.m. route with two doses (2×10^6^ CFU/100 μl) of the Δ*lpp* Δ*msbB* Δ*ail*, Δ*lpp* Δ*msbB*::*ailL2* or the Δ*lpp* Δ*msbB* Δ*pla* mutant. One group of mice received PBS *in lieu* of bacteria, thus representing a naive, unimmunized control. Two doses of the vaccine were given in a 50 μl volume in each of the hind legs 21 days apart.^25^ Animals were assessed for morbidity and/or mortality over the duration of vaccination.

#### Antibody production

Retro-orbital bleeding of all mice occurred on days 0, 14, 35, 56, 81 and 112. Sera were filtered by using Costar 0.1-μm centrifuge tube filters (Corning Inc., Corning, NY, USA). ELISA plates were coated with the F1-V fusion protein (1 ng/ml, BEI Resources, Manassas, VA, USA) overnight at 4 °C.^23,24,26^ Total IgG and antibody isotypes (IgG1, IgG2a and IgG2b) against F1-V in the sera (1:5 serially diluted) of all mice were determined as we previously described.^23,25^

#### Organ harvesting

To evaluate immunogenicity of the mutant strains, spleens from three control and five immunised mice (per group per time point) were isolated on days 42, 63 and 84. In addition, another set of mice (immunised and control, *n*=8–10 per group) were first exposed to 1.2×10^4^ CFU/40 μl (24 LD_50_) of the bioluminescent WT *Y. pestis* CO92 *luc2* strain on day 120 after the first immunisation. Spleens were then isolated from these surviving mice (*n*=3–5 per group) on either day 124 (4 days p.i.) or on day 141 (21 days p.i.). The PBS-injected mice without exposure to WT CO92 *luc2* (*n*=3) served as an additional control for the T cell experiment. Single cell suspensions were prepared by forcing the spleens through nylon cell strainers and by suspending the cells in RPMI 1640 medium with 10% fetal bovine serum. Cells were collected by centrifugation, with blood cells being removed by using red blood cell lysis buffer (Sigma-Aldrich; St Louis, MO, USA).

#### Flow cytometry analysis

All cells were stained with Ghost Dye-APC/Cy7 (Tonbo biosciences; San Diego, CA, USA) to gate for live cell populations. Splenic cells (1×10^6^) were incubated with 0.5 μg/sample anti-mouse CD16/32 antibody (BioLegend; San Diego, CA, USA) for 10 min on ice to prevent non-specific binding of monoclonal antibodies to the Fc receptors. The surface of the B cells was stained with monoclonal anti-mouse CD19-FITC (B-cell surface marker; BioLegend), anti-mouse CD38-PE/Cy7 (memory B-cell marker; BioLegend) and anti-mouse IgG-PE (mature, isotype-switched B-cell marker; Southern Biotech; Birmingham, AL) for 30 min in the dark at 4 °C.

To measure T cell kinetics, splenic cells were pretreated with ionomycin (750 ng/ml) and phorbol 12- myristate 13-acetate (PMA, 50 ng per sample), and then incubated 2 h later with Brefeldin A (0.7 μg per sample) to accumulate intracellular cytokines. The surface of the T cells was stained with monoclonal anti-mouse CD4-PE/Dazzle594 (BioLegend) and anti-mouse CD8-FITC (BioLegend) for 30 min in the dark at 4 °C to distinguish CD4^+^ and CD8^−^ expressing cells. Stained cells were centrifuged, washed and permeabilized with Foxp3 staining buffer set (eBioscience; San Diego, CA, USA). T cells were stained with anti-mouse IFN-γ-Percp/Cy5.5 (BioLegend), anti-mouse IL-17A-PE/Cy7 (BioLegend) and anti-mouse Foxp3-PacificBlue (eBioscience) for 30 min in the dark at 4 °C.

To determine innate immune responses, the surface of DCs was stained with monoclonal anti-mouse CD11c-PE/Dazzle594 (BioLegend), anti-mouse CD11b-Percp/Cy5.5 (BioLegend), anti-mouse CD80-Pacific Blue (BioLegend), anti-mouse CD86-FITC (BioLegend) and anti-mouse MHC-II-PE (Tonbo Biosciences) for 30 min in the dark at 4 °C.

All stained cells were fixed with 1% paraformaldehyde in PBS, examined for sterility and then subjected to flow cytometry. Suitable isotype antibodies for all experiments were used as controls. The differential cell population was acquired on flow cytometer (LSRII Fortessa) and analysed using FACS diva software (BD Biosciences, San Jose, CA, USA).

#### Challenge

On day 120, a set of immunised and PBS-injected control mice (*n*=5 per group) were exposed by the i.n. route with 1.2×10^4^ CFU/40 μl (24 LD_50_) of the bioluminescent WT *Y. pestis* CO92 *luc2* strain, which contains the luciferase operon (*luc*), allowing *in vivo* imaging of mice for bacterial dissemination in real time.^23^ On days 123 and 130 (day 3 and 10 p.i.), the animals were imaged by using an *in vivo* imaging system (IVIS) 200 bioluminescent and fluorescence whole-body imaging workstation (Caliper Corp.; Alameda, CA, USA) in the ABSL-3 facility to examine dissemination and progress of infection.

#### Rat studies

Rats were immunised by the i.m. route with two doses (2×10^6^ CFU/100 μl, 21 days apart) of the Δ*lpp* Δ*msbB* Δ*ail* or the Δ*lpp* Δ*msbB* Δ*pla* mutant. One group of rats received PBS *in lieu* of bacteria, thus representing a naive, unimmunized control group. Saphenous vein bleeding of all rats occurred on days 0, 14, 35, 56, 77 and 88. Sera were processed as described above for mice. Total IgG against F1-V in the sera (1:5 serially diluted) of all rats was determined as we previously described.^23,25^

On day 42, spleens were isolated from rats (*n*=3 per group per time point) and were pretreated with ionomycin and PMA and then incubated 2 h later with Brefeldin A. The surface of the T cells was stained with monoclonal anti-rat CD4-PE/Cy7 (BioLegend) and anti-rat CD8-APC (BioLegend) for 30 min in the dark at 4 °C to distinguish CD4^+^ and CD8^−^ expressing cells. Stained cells were centrifuged, washed and permeabilized with Foxp3 staining buffer set (eBioscience). T cells were stained with anti-rat IFN-γ-PE(BioLegend) and anti-rat IL-17A-Percp/Cy5.5 (eBioscience) for 30 min in the dark at 4 °C. Cells were analysed by Flow Cytometry as described above.

Another set of immunised and PBS-injected control rats (*n*=6 per group) were exposed by the i.n. route with 1.6–2.3×10^4^ CFU/50 μl (31–46 LD_50_) on day 43 or day 91 of the WT CO92 *luc2* strain. Animals were assessed for morbidity and/or mortality for 2–3 weeks after the exposure. During the second challenge (on day 91), blood and organs (lungs, liver and the spleen) from surviving rats were collected on day 14 post challenge. An aliquot (200 μl) of the blood and homogenised organs were plated on the blood agar plates to examine the clearance of the challenge strain from these rats.

### Statistical analysis

For majority of the experiments, one-way analysis of variance (ANOVA) and two-way ANOVA were used with the Tukey's *post hoc* test for data analysis. We used Kaplan–Meier survival estimates with log-rank (Mantel–Cox) test for animal studies, and *P* values of ⩽0.05 were considered significant for all of the statistical tests used.
